# Genome-wide identification and analysis of monocot-specific chimeric jacalins (MCJ) genes in Maize (*Zea mays* L.)

**DOI:** 10.1186/s12870-024-05354-4

**Published:** 2024-07-06

**Authors:** Hailong Jiang, Jiajian Peng, Qian Li, Siqian Geng, Hualei Zhang, Yuting Shu, Rui Wang, Bin Zhang, Changsheng Li, Xiaoli Xiang

**Affiliations:** The National Engineering Laboratory of Crop Stress Resistance Breeding, Anhui Agricultural University, Hefei, China

**Keywords:** Maize, Jacalin and dirigent proteins, Phylogenetic analysis, Subcellular localization, Agglutination activity

## Abstract

**Background:**

The monocot chimeric jacalins (MCJ) proteins, which contain a jacalin-related lectin (JRL) domain and a dirigent domain (DIR), are specific to Poaceae. MCJ gene family is reported to play an important role in growth, development and stress response. However, their roles in maize have not been thoroughly investigated.

**Results:**

In this study, eight *MCJ* genes in the maize genome (designated as *ZmMCJs*) were identified, which displayed unequal distribution across four chromosomes. Phylogenetic relationships between the ZmMCJs were evident through the identification of highly conserved motifs and gene structures. Analysis of transcriptome data revealed distinct expression patterns among the *ZmMCJ* genes, leading to their classification into four different modules, which were subsequently validated using RT-qPCR. Protein structures of the same module are found to be relatively similar. Subcellular localization experiments indicated that the ZmMCJs are mainly located on the cell membrane. Additionally, hemagglutination and inhibition experiments show that only part of the ZmMCJs protein has lectin activity, which is mediated by the JRL structure, and belongs to the mannose-binding type. The *cis*-acting elements in the promoter region of *ZmMCJ* genes predicted their involvement response to phytohormones, such as abscisic acid and jasmonic acid. This suggests that *ZmMCJ* genes may play a significant role in both biotic and abiotic stress responses.

**Conclusions:**

Overall, this study adds new insights into our understanding of the gene-protein architecture, evolutionary characteristics, expression profiles, and potential functions of *MCJ* genes in maize.

**Supplementary Information:**

The online version contains supplementary material available at 10.1186/s12870-024-05354-4.

## Background

Lectins are a group of glycoproteins that are not capable of causing an immune response [1]. They harbor at least one reversible non-catalytic site that binds to sugars, and play important roles in various cellular processes such as, cell division, endogenous regulation [2], and responses to stress conditions [3].

Jacalin-related lectins (JRLs) are carbohydrate-binding proteins, which are lectins isolated from jackfruit [4]. In recent times, JRLs have emerged as a new subcategory of non-traditional lectins, and that are involved in response to both biotic and abiotic stressors [5]. Based on their ability to bind to sugars, the JRL family can be divided into two main types: galactose-binding lectins (storage lectins found in vesicles) [6], and mannose-binding lectins (inducible lectins found in the cell nucleus and cytoplasm) [7]. The mannose-binding lectins may play a widespread role in intracellular signal transduction [8, 9]. From a structural perspective, these lectins can be classified into three categories: 1) lectins with only jacalin domain, 2) hybrid lectins (contains jacalin domain and another type of domain viz. Dirigent (DIR and DIR-like), NB-ARC (nucleotide-binding domain shared with APAF-1, various R-proteins and CED-4), F-Box (a diverse class of adaptor proteins of the ubiquitin–proteasome system), GNA (Galanthus nivalis agglutinin), or PAG (polynucleotide adenosine glycosidase) [10], and 3) total lectins with multiple concatenated jacalin domains [11]. Hybrid lectins have become the focus of research due to their unique combinations of structural domains, which may give them functionalities that are not seen in their parent genes. This can often be a driving force behind adaptive evolution [12].

The unique lectin subfamily found in monocotyledonous plants, known as the *MCJs* (Dirigent and Jacalin fusion lectin) gene family, is formed through the fusion of Jacalin and Dirigent proteins [13]. Previous reports in rice and wheat suggests that the primary function of the *MCJs* family is defense against both biotic and abiotic stressors [14]. The Dirigent domain belongs to the DIR gene family, primarily involved in the biosynthesis of lignin and lignans [15]. It also regulates cell wall metabolism and is responds to various stressors, including infestations by pests [16, 17]. Presently, a total of 11 *MCJs* have been identified, including Beta-Glucosidase Aggregation Factor 1 (*BGAF1*) in maize and sorghum [18, 19], *HvJPR1* (*32* kDa* protein JRG1.2 gene, jasmonate-regulated*) in barley [20], *OsJAC1* (*MANNOSE-SPEDCIFIC JACALIN-RELATED LECTIN*) in rice [21], *Crs-1* (*creeping specific-1*) in bent grass [22], as well as *TaJA1* (*JASMONATE-INDUCED PROTEIN1*), *TaVer2* (*VERNALIZATION2*), *TaWCI-1* (*WHEAT CHEMICALLY INDUCED 1*), *TaHfr* (*HESSIAN FLY-RESPONSIVE DISEASE RESISTENCE*), *TaMCJ1* (*Monocot chimeric* *jacalin 1*) and *TaMCJ3* (*Monocot chimeric* *jacalin* *31*) in wheat [14, 23, 24]. These MCJs share remarkable structural similarities and play vital roles in plant development and response to stressors [25].

In rice, *OsJAC1* overexpression enhances resistance against non-host pathogenic microorganisms, wherein OsJAC1 accumulates at the fungal infection sites [26], While the JRL domain of OsJAC1 is involved in the recognition of the mannose complexes derived from fungal hyphae, the DIR domain recruits other defense-related proteins to form disease resistance complexes, which inhibits the pathogens [27]. Similarly, barley *TaJA1* overexpression in tobacco enhances resistance against tobacco powdery mildew [28] and fungal pathogens in barley [29]. Wheat *TaMCJ1* and *TaMCJ3* overexpression results in enhanced resistance to wildfire disease, and dehydration tolerance, respectively [14]. The maize *ZmBGAF1* (homodimer, aggregates beta-glucosidases) forms chain-like complexes, associated with pest resistance [30, 31]. The primary function of beta-glucosidases is to release toxic glycoside elements (e.g., DIMBOA,) that contributes to resistance against pests including aphid and corn borer [32].

Maize (*Zea* *mays*, family: Poaceae) is an important cereal worldwide [33]. This study presents the identification of MCJ gene family members in maize genome, followed by analysis of their gene structure, sequence characteristics, evolutionary relationships, and subcellular localization. Additionally, predictive protein–protein interaction networks of *ZmMCJ* gene members have been examined. The expression profiles *ZmMCJ* members have been examined across various tissues, using publicly available transcriptome sequencing data as well as by Reverse Transcription-quantitative Polymerase Chain Reaction (RT-qPCR) analysis. Blood agglutination and inhibition experiments were also carried out to investigate the lectin functions of ZmMCJ members and their potential substrates. This research sets the groundwork for further exploration of the role of the *ZmMCJ* gene family in growth and development.

## Results

### Identification of *ZmMCJ* gene family members in the maize genome

**Fig. 1:**
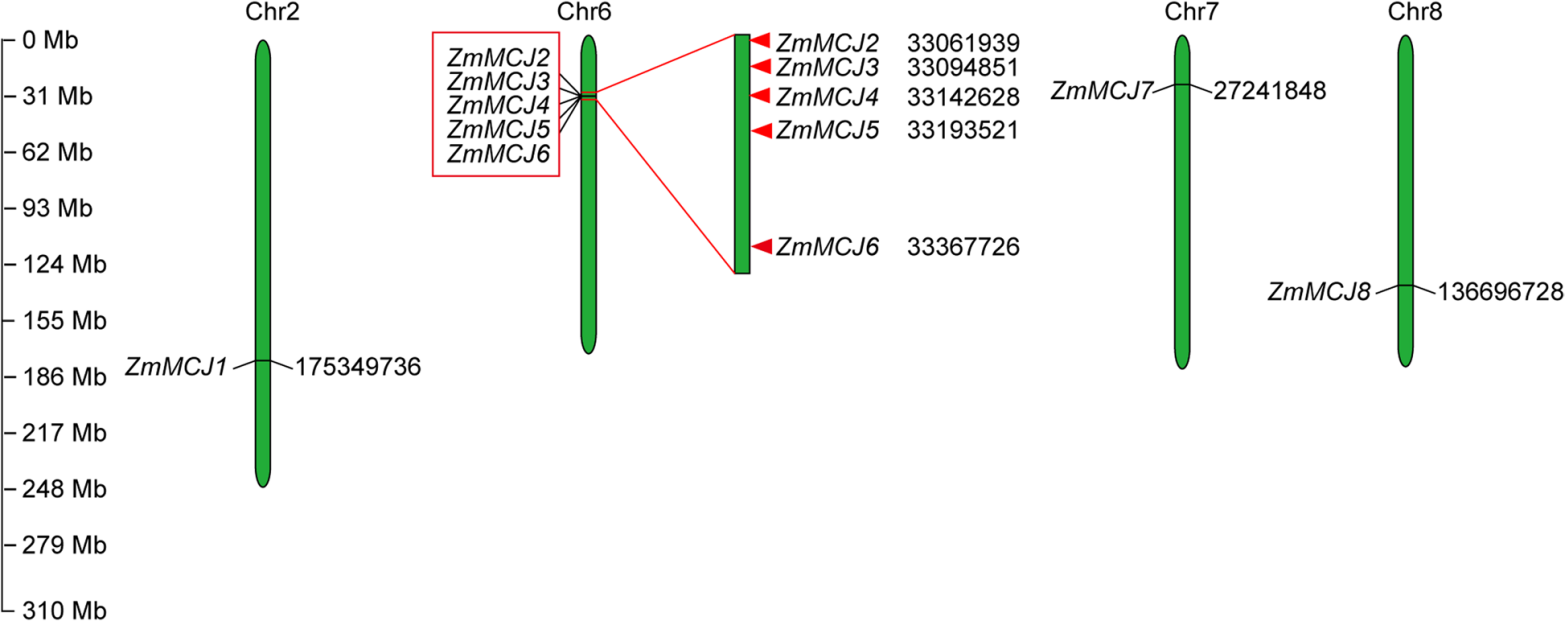
Chromosomal locations of eight *ZmMCJs* in maize. The read box on chr.6 indicated the tandem duplication event

To characterize the number of MCJ family members, we searched the maize MCJ proteins in the Ensembl and Phytozome databases, which were both based on the B73 genome version 4. This search resulted in the identification of eight *ZmMCJ* members with minor variations in predicted characteristics (Supplementary Table S1). The *ZmMCJ* genes are unevenly distributed on four chromosomes, with chromosome no. 6 bearing five members (Fig. 1).

### Phylogenetic relationships and gene structure analysis of ZmMCJ family members

**Fig. 2:**
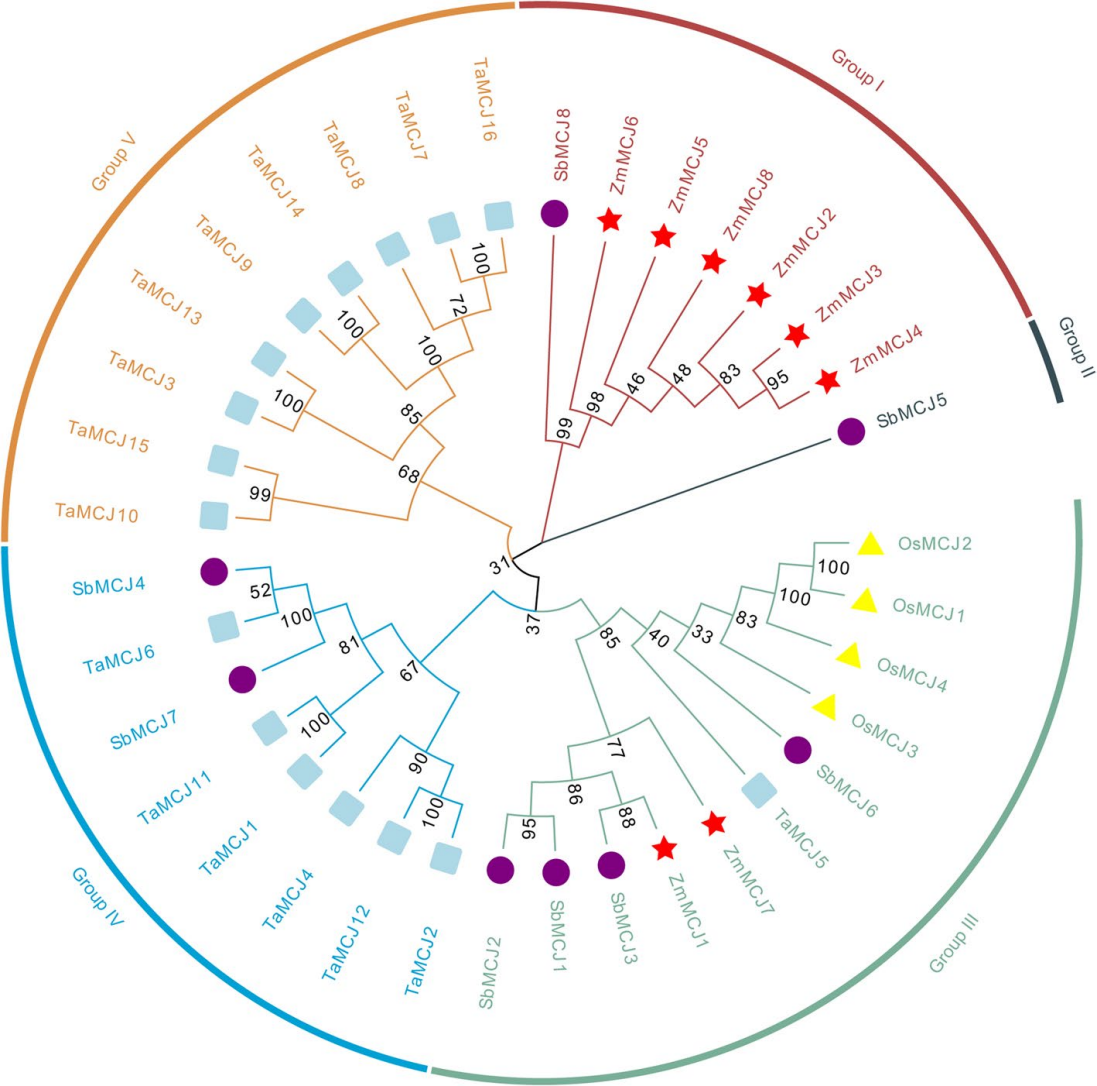
Phylogenetic relationship of *ZmMCJs*, *OsMCJs*, *TaMCJs* and *SbMCJs*. An un-rooted phylogenic tree was constructed in MEGA11 based on multiple alignment of full sequences from four monocot species (*Z. mays, O. sativa, T. aestivum* and *S. bicolor*)

**Fig. 3:**
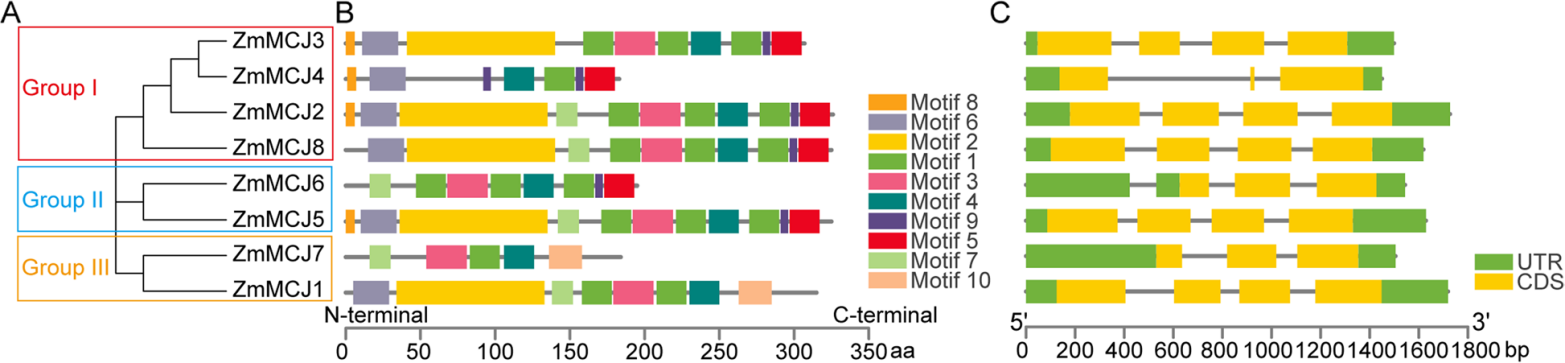
Motif structure and gene structure, analysis of *ZmMCJs*. **A** Phylogenetic tree of ZmMCJ proteins. **B** 10 conserved motifs were identified in protein sequences of ZmMCJs. **C** Gene structures. Exons and introns were indicated by boxes and lines respectively

Phylogenetic analysis of 36 MCJ members, including members from Wheat (16 members), Rice (4 members), Sorghum (8 members), and Maize (8 members) was carried out (Fig. 2). The MCJ proteins were classified into five Groups (I to V), wherein the eight ZmMCJ members were placed in Group I and Group III. Notably, the maize MCJ proteins exhibited a prominent presence in Group I, implying a high level of similarity for these *MCJ* genes. Furthermore, two members (ZmMCJ1, ZmMCJ7) were clustered with the MCJ proteins from wheat, rice, and sorghum, in Group III (Fig. 2). The eight maize ZmMCJs could be further sub-categorized into Groups I to III (Fig. 3A). Through protein motif analysis of the ZmMCJ members, we identified the presence of ten conserved motifs (Fig. 3B and Fig. S1). All ZmMCJ proteins contained motif1 and motif4, with most of the members also featuring motif2, motif3, motif5, motif6, motif7, and motif9. Except for ZmMCJ4 in subgroup I, all proteins included motif3. Interestingly, only ZmMCJ1 and ZmMCJ7 of subgroup III possessed motif10. In terms of the gene structures, the ZmMCJs in subgroup I typically comprised of 4 exons, except for ZmMCJ4 which consisted of 3 exons. On the other hand, ZmMCJ6 of subgroup II contained 3 exons, while ZmMCJ5 featured 4 exons. In subgroup III, ZmMCJ7 contained 3 exons, whereas ZmMCJ1 consisted of 4 exons (Fig. 3C).

### Synteny analysis of the *ZmMCJ* gene family

**Fig. 4:**
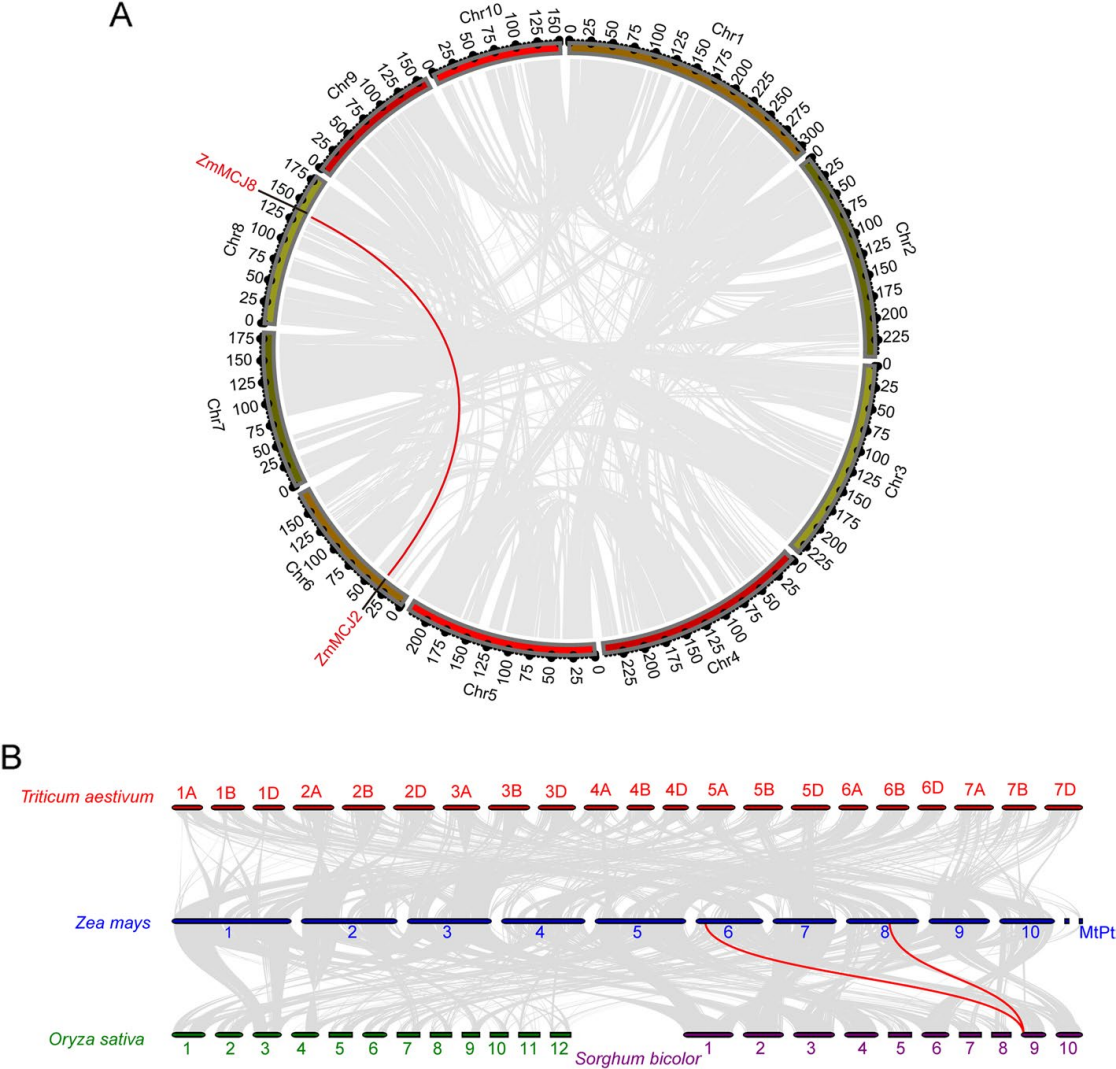
Schematic diagram of the syntenic relationships of ZmMCJs. **A** Schematic diagram of the syntenic relationships of ZmMCJs in maize. The gray ribbons represent syntenic blocks in the Maize genome, and the segmental duplication events are marked in red. **B** Synteny analysis of MCJ genes among Wheat, Maize, Rice, and Sorghum. Gray lines in the background indicate collinear blocks within Maize and the indicated plant species, whereas the red lines highlight syntenic MCJ gene pairs

In order to investigate the evolution of the *ZmMCJ* gene family in maize, distribution of the eight identified ZmMCJ genes on the 10 maize chromosomes and segmental and tandem duplication events were analyzed. Homologs located on different chromosomes were considered as segmental duplications. Among the eight *ZmMCJ* genes, one set of tandem duplication event, namely *ZmMCJ2*,* ZmMCJ3 ZmMCJ4*, *ZmMCJ5* and *ZmMCJ6*, was detected (Fig. 1). One set of segmental duplicates, namely *ZmMCJ2* and *ZmMCJ8*, were detected (Fig. 4A). The Ka/Ks ratio range from 0.08 to 0.60 suggested the presence of purifying selective pressure during *ZmMCJs* gene family evolution, indicating a potential shared conserved function among these genes. But the *ZmMCJ* genes vary in gene-protein organization, suggesting some differences also in the roles. Furthermore, a comparative syntenic map for insights into relationship between the maize genome and the genomes of Wheat, Rice, and Sorghum (Fig. 4B), displayed collinearity of two maize *ZmMCJ* genes (*ZmMCJ2* and *ZmMCJ8*) with one Sorghum *SbMCJ* gene (*SbMCJ5*), suggesting potential significance of these orthologous pairs in plant evolution. These genes are all in Group I (Fig. 2), suggests they may have similar functions.

### Identification of stress-related *cis*-acting elements in the* ZmMCJ* gene promoters

The outcome of analysis of *cis*-acting elements among eight *ZmMCJ* genes exhibits presence elements associated with response to abscisic acid (ABRE, ABRE3a, ABRE4) and MeJA (CGTCA-motif) (Fig. S3). The promoter regions of eight *ZmMCJ* genes contain numerous anaerobic response elements (ARE), and most of them possess the drought-inducibility element MBS, suggesting MYB transcription factor-mediated regulation. Multiple gene promoters also harbored low temperature response element (LTR) and, four *ZmMCJ* gene promoters encompass O2 binding sites, indicating their potential significance in maize endosperm development.

### Tissue and developmental stage specific expression pattern of *ZmMCJs*

**Fig. 5:**
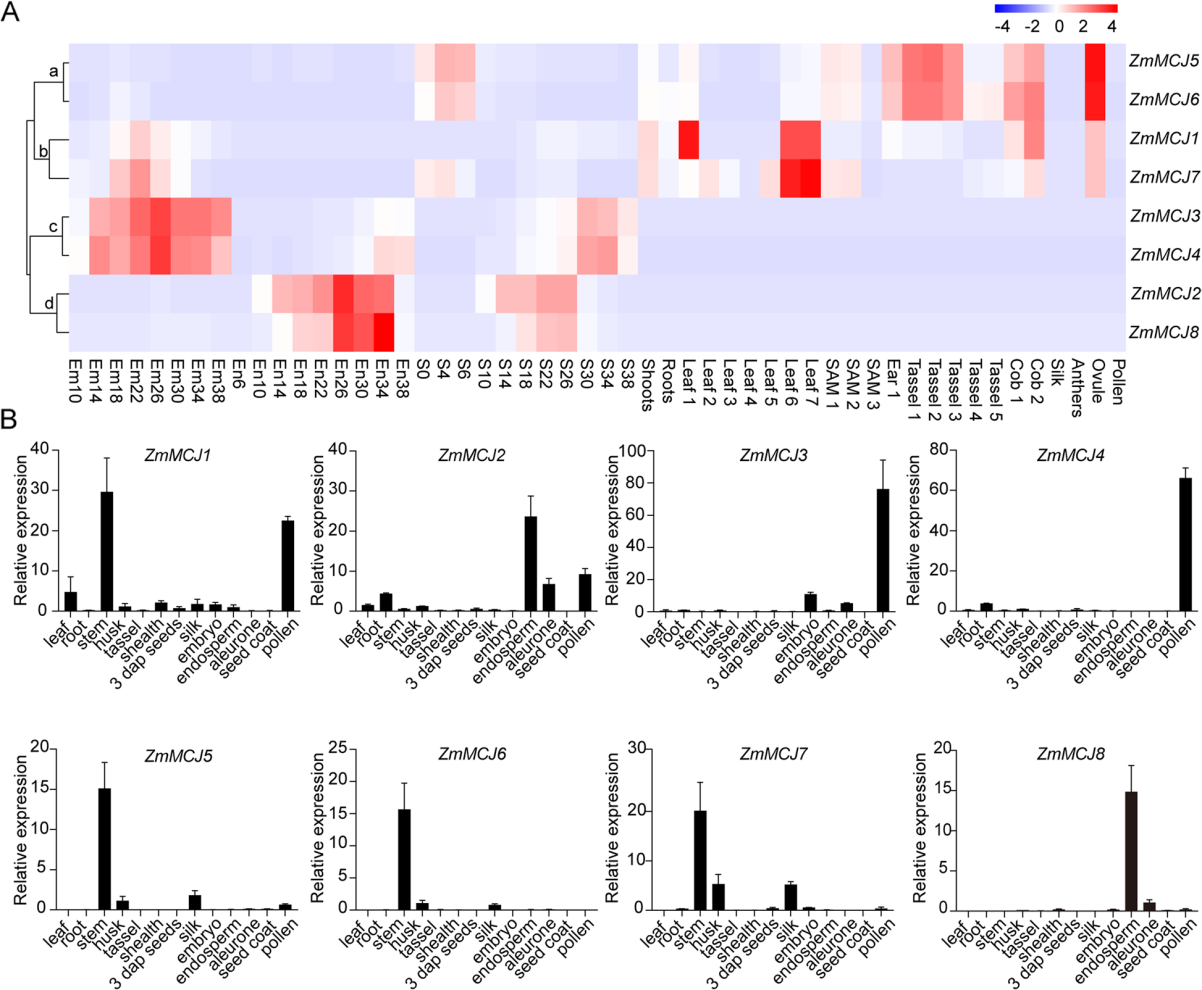
The expression profiles of *ZmMCJs* genes in maize. A Heat map of *ZmMCJs* genes expression in different tissues and developmental stages. The genes are located on the right, and the tissues are indicated at the bottom of each column. The color bar represents the expression values. S0-S38: developing seed from 0 to 38 DAP (day after pollination); Em10-Em38: developing embryo from 10 to 38 DAP; En10-En38: developing endosperm from 10 to 38 DAP. S0-S38: developing seeds from 0 to 38 DAP. **B** Results of quantitative RT-PCR of eight *ZmMCJ* gens in different tissues. *Zmactin* was used as the internal reference gene. Data were shown as mean ± s.d., *n* = 3 biological replicates; each biological replicate represents an individual plant. Statistically significant differences are indicated (*p* < 0.05, Student’s *t*-test)

By analyzing the transcript data obtained from public RNA-seq data [21], the tissue and developmental stage specific expression patterns of the *ZmMCJ* genes was analyzed (Fig. 5A). The *ZmMCJs* were clustered into four main modules (a to d) based on expression abundance. Module a genes (*ZmMCJ5*, *ZmMCJ6*) showed high expression levels in ovule, tassel1, tassel2, tassel3, cob1, cob2, ear, and early-stage seeds (S0, S4, S6), while module b genes (*ZmMCJ1*, *ZmMCJ7*) exhibited high levels in leaf 6 and leaf 7, with *ZmMCJ1* also being highly expressed in leaf 1. Module c genes (*ZmMCJ3*, *ZmMCJ4*) displayed embryo-specific patterns with higher expression level, while module d genes (*ZmMCJ2*, *ZmMCJ8*) were expressed solely in the endosperm. *ZmMCJ2* starts expressing at 10 DAPS (days after pollination), which is the initiation stage of endosperm filling, while* ZmMCJ8* starts expressing during the middle stage of endosperm filling (14 DAP). Both *ZmMCJ2* and *ZmMCJ8* are highly enriched throughout endosperm development.

Validation of expression profiles of eight *ZmMCJs* (from module a to c) by RT-qPCR was done in multiple plant organs (including leaves, roots, stems, husks, tassels, sheaths, 3 DAY seeds, silks, embryos, endosperms, aleurones, seed coats, and pollen) showed consistency in some genes with the RNA-seq data (Fig. 5B). The expression levels of two genes (module a: *ZmMCJ5* and *ZmMCJ6*) from was prominent in stems, husks, and silk. Two module b genes (*ZmMCJ1* and *ZmMCJ7*) exhibit high expression levels in stems. Two module c (*ZmMCJ3* and *ZmMCJ4*) showed high expression in pollen. Two others module d (*ZmMCJ2* and* ZmMCJ8*) showed high expression in endosperm.

### Subcellular localization of ZmMCJs

**Fig. 6:**
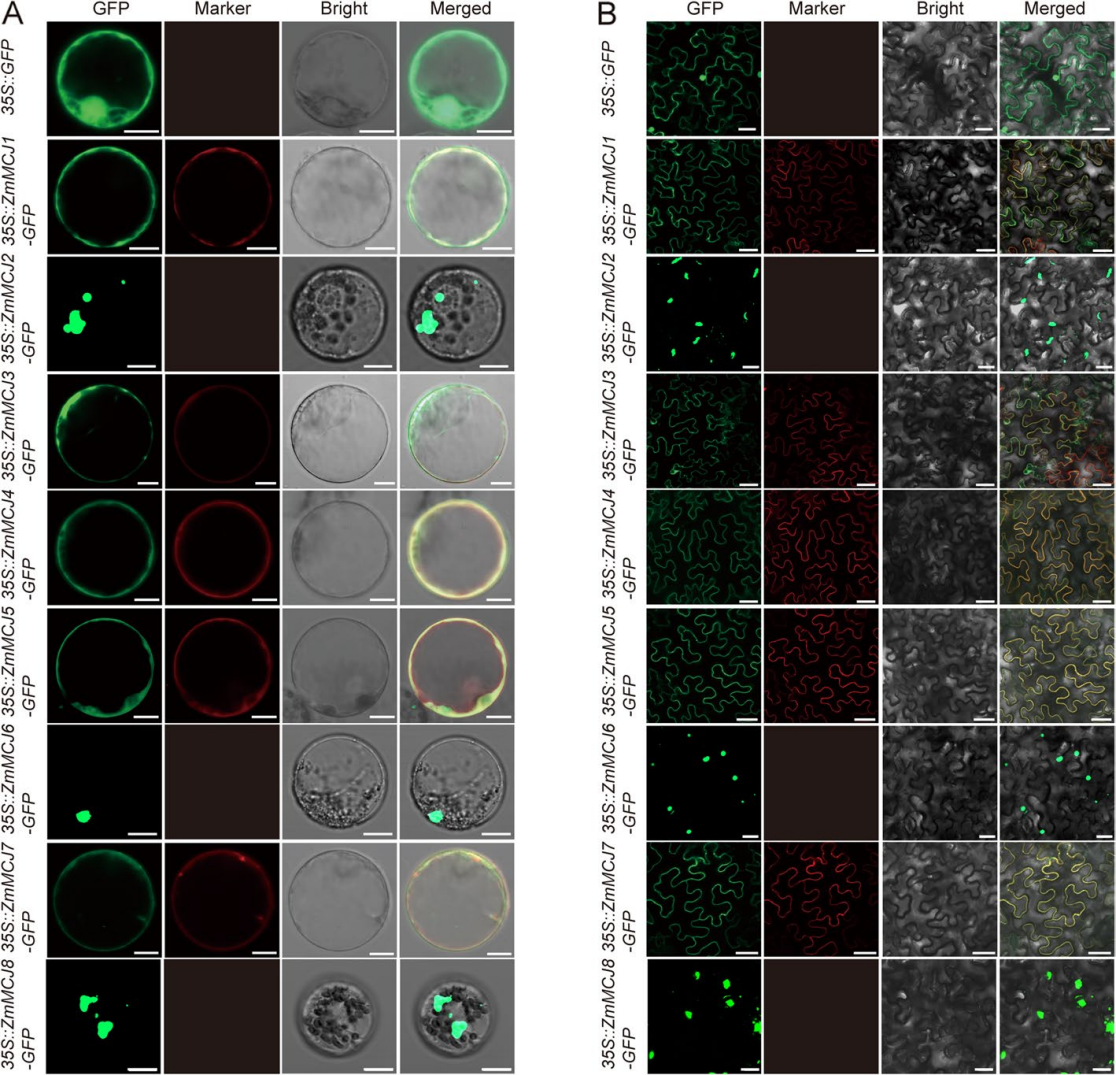
Subcellular localization of eight ZmMCJs. **A** The constructs were transiently expressed in maize leaves protoplast. **B** The constructs were transiently expressed in *N. benthamiana* leaves via *Agrobacteria* infiltration. The *ZmMCJ* genes were cloned from maize inbred line B73 and used to construct CaMV35S::*ZmMCJs*-GFP vectors in which GFP was fused at the C-terminus. The mCherry-*OsRAC1* was used as cell membrane marker. Pictures were taken using a confocal fluorescence microscope. Scale bars = 50 μm

To determine the subcellular localization of eight ZmMCJ members, each selected MCJ protein was fused to green fluorescent protein (GFP). The free GFP was used as the control. The constitutive 35S promoter drove all gene cassettes. We transiently expressed the ZmMCJ-GFP fusion proteins in both maize yellow seedling protoplast (Fig. 6A) and tobacco leaves (Fig. 6B). All signals of the fused proteins except *35S::ZmMCJ2:GFP,* *35S::ZmMCJ6:GFP, 35S::ZmMCJ8:GFP* were localized in cell membrane, whereas the control *35S:GFP* was detected both in nuclei and the cytoplasm (Fig. 6A, B). *35S::ZmMCJ2:GFP,* *35S::ZmMCJ6:GFP, 35S::ZmMCJ8:GFP* were localized as dot signal in cytoplasmic (Fig. 6A, B). They formed numerous small droplets of various sizes, which is a phenomenon characteristic of liquid–liquid phase separation [34]. These signals also similar to two Metacaspases (ZmMC1 and ZmMC2), which are mainly localized in punctate dots aggregates and are partially co-localized with autophagosomes [35]. Whether ZmMCJ2, ZmMCJ6 and ZmMCJ8 are liquid–liquid phase separation or autophagosome related, further study needs to be done.

### Analysis of protein tertiary structures

**Fig. 7:**
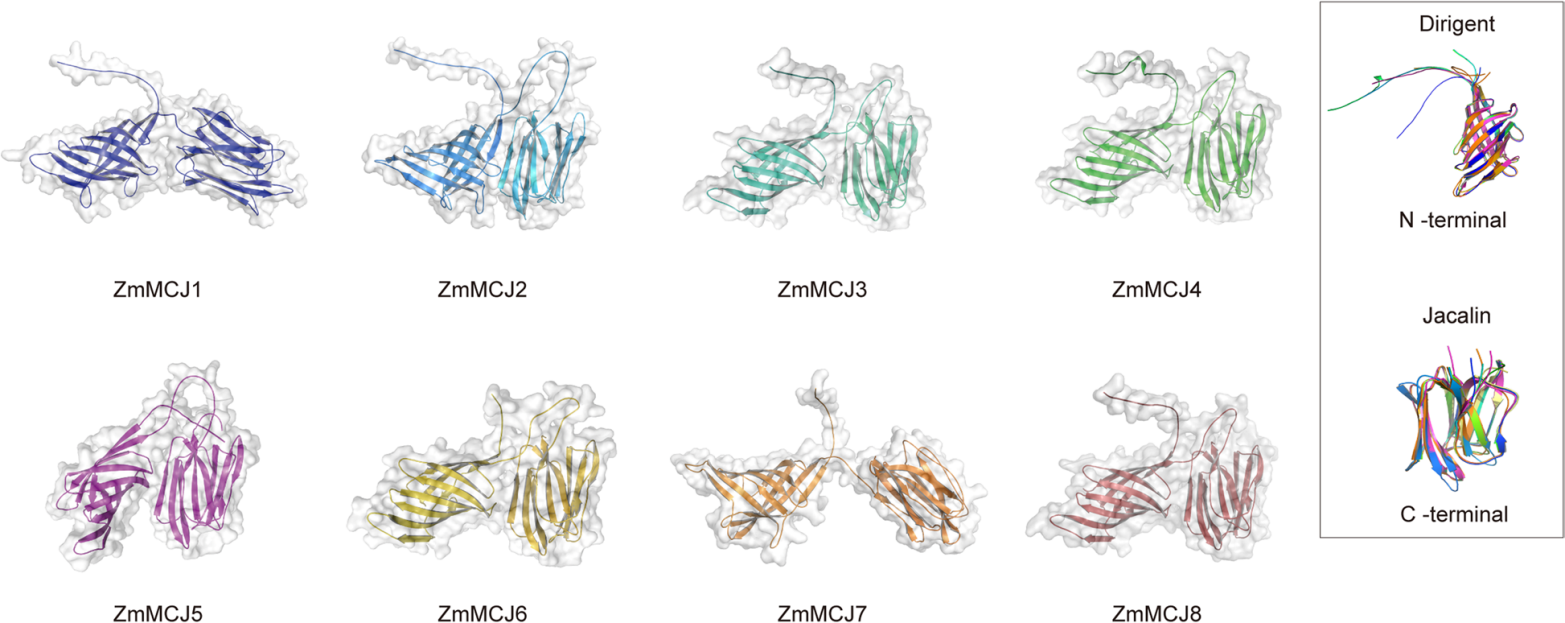
Predicated structures of eight ZmMCJ proteins. Dirigent domain is on the N-terminal of MCJ proteins. Jacalin domain is on the C-terminal of MCJ proteins

The ZmMCJ protein consists of the JRL domain and the DIR domain. Both domains are highly conserved, predominantly comprising β-sheets, β-turns, and minor irregular coils. The 3D structures of the ZmMCJ1/7, ZmMCJ2/8, ZmMCJ3/4 and ZmMCJ5/6 proteins were similar to each other respectively, interestingly, this fits with their organizational positioning (Fig. 7).

### Agglutination activity and carbohydrate-binding properties

**Fig. 8:**
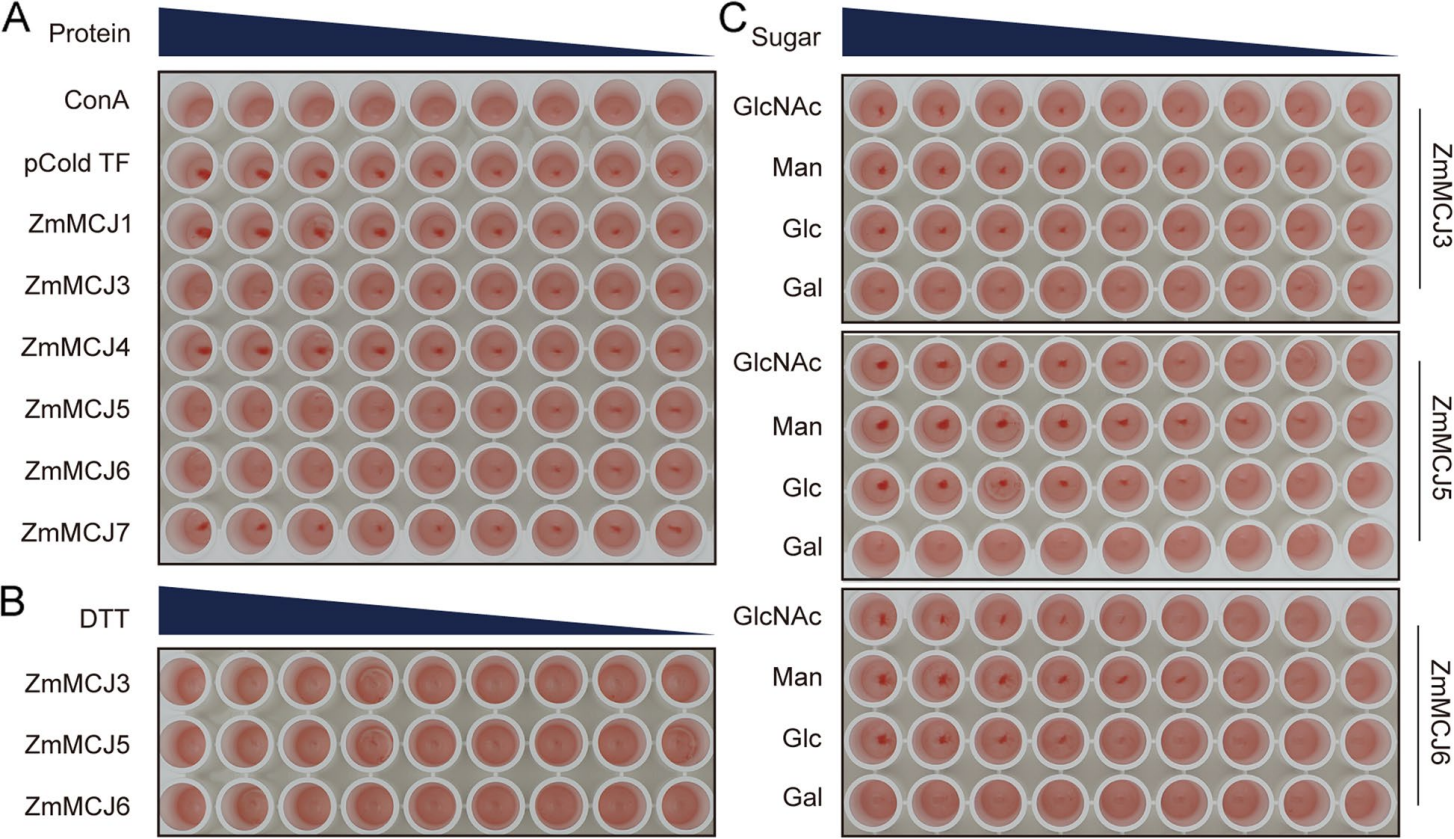
Agglutination activity and carbohydrate-binding properties of TF-ZmMCJs. **A** Agglutination activity test. The initial concentration of ConA, TF and TF-ZmMCJs is 0.5 mg/mL, and it is diluted 2 times each time. **B** The initial concentration of DTT is 2 mM, and it is diluted 2 times each time, The concentration of TF-ZmMCJs is 0.1 mg/mL.** C** Carbohydrate-binding property test: GlcNAc, Gal, Glc and Man as inhibitors (final concentration approximately 66.7 mM); the carbohydrate-binding specificities of TF-ZmMCJ3 was inhibited by Man when diluted in 192-fold increments, by Glc in 192-fold increments, by Gal or GlcNAc in 768-fold increments

In order to explore the agglutinin function of ZmMCJ proteins, the purified proteins were used to conduct cell agglutination tests based on rabbit red blood cells. The concentration of ConA, TF, and TF-ZmMCJs was 0.5 mg/mL. ConA had the ability to clump together rabbit red blood cells, whereas TF did not. This outcome confirms the success of the tests. Approximately 0.0625 mg/ml of TF-ZmMCJ3 was able to cause agglutination of rabbit erythrocytes, while approximately 0.0020 mg/ml of either TF-ZmMCJ5 or TF-ZmMCJ6 also resulted in agglutination of rabbit erythrocytes. Additionally, approximately 0.98 mg/ml of ConA induced the same effect (Fig. 8A). On the other hand, TF-ZmMCJ1, TF-ZmMCJ4, and TF-ZmMCJ7 did not lead to clumping of rabbit red blood cells (Fig. 8A). Analysis of carbohydrate-binding specificities of TF-ZmMCJs, inhibition assays were conducted using rabbit red blood cells. The highest concentration of DTT allowed inhibition of TF-ZmMCJ3, TF-ZmMCJ5, and TF-ZmMCJ6, as shown in Fig. 8B. Furthermore, the minimum inhibitory concentrations of GlcNAc, Man, and Gac for TF-ZmMCJ3 were 0.0010, 0.0005, and 0.0010 mg/ml, respectively (Fig. 8C). Therefore, Man was the most effective inhibitor for TF-ZmMCJ3. For TF-ZmMCJ5, the minimum inhibitory concentrations of GlcNAc, Man, and Glc were 8, 4, and 8 times higher, respectively. Among them, Man had the lowest concentration. Overall, TF-ZmMCJ3, TF-ZmMCJ5, and TF-ZmMCJ6 demonstrated the highest affinity for Man and were unaffected by Gal. These lectins (ZmMCJ3, ZmMCJ5, and ZmMCJ6) belong to a group known as mannose-binding jacalin-related lectins [36].

## Discussion

In recent years, numerous studies have focused on the analysis of MCJ gene family in plant species. These genes play various roles in plant growth, development, and response to stress [25, 37]. In this study, eight MCJ family members were identified in maize, which is two times of rice (four) and Moso bamboo (*Phyllostachys* *edulis*) [5], half of wheat (16), and the same as sorghum (eight) [29]. This suggests that certain MCJ family members have been relatively conserved throughout evolution. Most of *ZmMCJ* genes were located as a gene cluster on chromosome 6. This suggests that duplication events may have occurred more frequently on chromosome 6 during the evolution of gene families, possibly influencing their functions [38, 39].

A previous study also constructed a phylogenetic tree based on JRL proteins using the maximum likelihood method, which classified them into eight groups [37]. It implied that JRLs in maize were more closely related to sorghum, which was consistent with our study. The expansion of gene families and genetic evolution in plant genomes are influenced by fragment replication and tandem replication [40, 41]. Gene expression is regulated by *cis*-acting elements. Previous research has analyzed the *cis*-acting elements of *PeD-J* genes in bamboo [5]. Furthermore, it has been demonstrated that MCJs play a role in pest resistance [31], indicating their potential involvement in maize defense against pests and diseases [18]. Here, ZmMCJ2, ZmMCJ6 and ZmMCJ8 proteins were localized as dot signal in cytoplasmic. These might be liquid–liquid phase separation phenomenon, which might be involved in selective autophagy of invading pathogens [34, 42]. These signals are also like endosomes localized, which might have a novel role in defense response [35, 43]. The expression of *ZmMCJ* genes is tissue-specific in plant. Notably, *ZmMCJ3* and *ZmMCJ4* were high expressed in embryo, suggesting their importance in embryo growth and development. On the other hand, the expression of *ZmMCJ2* and *ZmMCJ8* gradually increased during the grain filling stage, which may be related to endosperm development. Lastly, the *ZmMCJ1* and *ZmMCJ7* genes exhibited high expression levels in leaves.

The JRL domain exhibits the common β-prism fold found in the JRL family, with the active site typically located within the concave cavity at the top of the prism [44]. It consists of three four-stranded β-sheets, forming three Greek key motifs [45]. The DIR domain adopts a β-barrel three-dimensional structure composed of eight antiparallel β-strands [46]. The 3D structure of ZmMCJs show that ZmMCJs might exist as heterodimer or homodimer in vivo [31].

Hemagglutination testing has been widely used for testing the lectin activity [21, 47]. Our data show that ZmMCJ3, ZmMCJ5 and ZmMCJ6 indeed exhibits the agglutination activity, whereas ZmMCJ1, ZmMCJ4, ZmMCJ7 do not have this function. The data suggest that ZmMCJ3, ZmMCJ5 and ZmMCJ6 are functional mannose-specific protein. They may play important role on autoimmune disease and against pathogens [36].

## Methods

### Identification of *ZmMCJ* genes in maize

The presence of MCJ genes in maize was investigated by HMMER3 [48] based on the search for dirigent domain (PF03018) and the jacalin domain (PF01419) in the maize genome database (https://www.maizegdb.org/). Hits with *E*-values less than or equal to 10^−10^ were considered as potential candidate for further analysis. Sequences containing both dirigent and jacalin domains (MCJ genes), were identified using Venn online software (http://bioinformatics.psb.ugent.be/webtools/Venn/) [49]. The MCJ genes were also searched in the genome databases of rice, wheat, and sorghum online at Ensembl (http://www.ensembl.org/index.html) and Phytozome (https://phytozome.jgi.doe.gov/pz/portal.html) databases. The basic physicochemical properties and signal peptide information of each MCJ family member was predicted using ExPASy ProtParam tool (https://web.expasy.org/protparam/). The MCJ genes identified were designated from *ZmMCJ1* to *ZmMCJ8*, based on their respective genomic locations within the maize chromosomes.

### Sequence alignment, phylogenetic relationships analysis

The ZmMCJ full-length protein sequences were aligned using the DNAMAN software version 10 (Lynnon Biosoft, San Ramon, CA, USA). To visually represent the conservation pattern of these sequences, sequence logos were created using the WebLogo tool [50]. For intraspecific phylogenetic trees (for relationships and similarity among different ZmMCJ full-length proteins) and interspecific phylogenetic trees (for relationships among MCJ protein domain sequences from different plant species), multiple sequence alignments were performed using Clustal W program using the default parameters [51] (gap opening penalty 10, gap extension penalty 0.2, gap distance 4, no end gaps and protein weight matrix using Gonnet). Further, the neighbor-joining (NJ) method in MEGA11 was employed to construct these phylogenetic trees [52]. The resulting trees were assessed for their confidence coefficients using 1,000 bootstraps with the pairwise deletion option.

### Chromosomal location and collinearity analyses

The details of physical position of the *ZmMCJ* genes were obtained from the maize genome database, while the block duplication events were visualized by TBtools [53]. For the construction of the syntenic map, *MCScanX* was employed [54], and the resulting map was visualized using TBtools. In *MCScanX*, tandem genes are assessed by gene rank according to chromosomal positions [54]. Using TBtools, the non-synonymous (Ka) and synonymous (Ks) substitution rates of the collinear gene pairs were estimated, for to predict the divergence time (t) and evolutionary rate (Ka/Ks ratio) [53].

### Motifs and gene structures of *ZmMCJ* genes

The MEME suite (http://meme-suite.org/) was utilized to detect and visualize the conserved patterns found in *ZmMCJ* genes, whereas TBtools software was used to analyze the gene structure, including introns and exons.

### Promoter motif analysis

To identify *cis*-acting elements in the promoter region, the 1,400 bp upstream sequence (preceding the ATG start site) of *ZmMCJ* genes was retrieved and analyzed at the PlantCare database (http://bioinformatics.psb.ugent.be/webtools/plantcare/html/), and the results were visualized using TBtools.

### Heat map analysis of RNA-Seq gene expression data

The maize reference genome B73 was utilized for analysis of the expression pattern of eight *ZmMCJ* genes [21]. R package 'heatmap', was used for generating a heat map and hierarchical clustering of *ZmMCJ* genes across 52 different samples (tissues and developmental stages). The gene expression values, normalized using log_2_ (RPKM + 1), were employed in this analysis. The sample IDs were assigned following the previously described procedure [21].

### Total RNA extraction and RT-qPCR analysis

Trizol reagent (Invitrogen Inc., Waltham, MA) was used to extract total RNA from maize tissues, and after *DNase I* digestion (Qiagen Inc., Germantown, MD, USA), the RNA was purified using the RNeasy Mini Kit. For endosperm RNA extraction, the same procedure as above, with the exception of using the RNA extract buffer prior to Trizol reagent [55]. Reverse transcription was conducted with ImProm-II™ Reverse Transcriptase (Promega, A3800). RT-qPCR analysis was performed with SYBR Green I using BIO-RAD CFX96 system. The PCR program included an initial step at 95 °C for 120 s, followed by 40 cycles of 95 °C for 5 s, 60 °C for 30 s, and 95 °C for 5 s. A melt curve analysis was performed from 65 °C to 95 °C, with an increment of 0.5 °C for 5 s. The 2^−∆∆Ct^ analysis method [56]was employed to analyze the expression levels of the target genes relative to the maize Actin gene (*ZmActin*, Zm00001d012277). The PCR primers used are listed in Table S3. Primers were designed by Primer Premier 5.0 to ensure primer specificity [57, 58].

### Subcellular localization analysis of ZmMCJ proteins

The ZmMCJ protein sequences were analyze by online tools like wolf-psort (https://psort.hgc.jp/) or PredictNLS (https://rostlab.org/owiki/index.php/ PredictNLS) to anticipate the nuclear localization signal (NLS). The C-terminal of each *ZmMCJ* was fused to an enhanced GFP (*eGFP*) in pCAMBIA2300 plasmid. Transformation of maize protoplasts involved polyethylene glycol (PEG)-mediated techniques, while Agrobacterium-mediated approaches were employed for tobacco (Nicotiana benthamiana) leaf epidermal cells. As a reference, a pCAMBIA2300-*GFP* vector encompassing a 35 S::*GFP* construct was used. For reliable results, a minimum of three replicates were used. A confocal microscope (Germany, Zeiss, LSM880) was used imaging of the eGFP signal.

### Homology modeling

The 3D structures of ZmMCJ proteins were predicted using homology modeling, which is based on their similarity to proteins that already have known structures. To find the appropriate templates for homology modeling, we retrieved them from the AlphaFold database (https://alphafold.ebi.ac.uk/).The template used for ZmMCJ1- ZmMCJ18 are B4G256, C0HFQ1, C0PDX9, B4F7S2、B4FV87, A0A1D6LHB1, B7ZZ04, C0PDX9, respectively. The EasyModeller software (http://softwaretopic.informer.com/easy-modeller-for-windows/) was used to model the main chain, side chain, and loop region of the proteins' tertiary structures, followed by refinement and optimization using the SAVES server (https://servicesn.mbi.ucla.edu/SAVES/). Visualization and manipulation of the predicted models was done using Discovery Studio, version 2016 (BIOVIA, Pairs, France).

### Purification of recombinant protein

To produce the recombinant TF -tagged proteins, the full-length CDS were amplified and inserted into cloning sites *Kpn I* and *EcoR I* in the pCold-TF expression vector (TAKARA) by homologous recombination, and used for overexpression in *Escherichia coli* DE3 (BL21) cells, induced by 1.0 mM IPTG at 16 6 lsessionat100 rpm/min. The cells were collected and sonicated, and the supernatants containing the corresponding fusion proteins were purified with NI-HA beads (QIAGEN). The proteins were eluted with 0 mM, 25 mM, 50 mM, 100 mM, 250 mM, 350 mM imidazole in 30 mM Tris–HCl buffer. The eluted proteins were analyzed on a 10% SDS-PAGE gel. The ZmMCJs proteins were eluted at 250 mM imidazole. The proteins were desalting concentration using Amicon Ultra 50 ml (UFC800308, Millipore, Merck).

### Agglutination activity and carbohydrate-binding property tests

Agglutination activity and carbohydrate-binding properties were tested using a two-fold serial dilution procedure [59]. ConA (*Canavalia ensiformis* lectin, which specifically binds mannose and glucose), and pCold TF empty vector (Trigger Factor (TF)-tagged protein) were used as positive and negative controls, respectively.

### Statistical analysis

The significance of difference between groups was analyzed by Independent Sample T Test analysis, and results presented as means ± standard deviation (± sd). Statistically significant differences were calculated by Student’s t test. Figures are made using software GraphPad Prism 9 and Adobe Illustrator 2024.

## Conclusions

A total of eight *ZmMCJ* genes were identified in the maize genome and were found to be distributed unevenly across four chromosomes, with highest number on Chromosome 6. Depending on the nature of the *cis*-acting elements, regulation of *ZmMCJ* genes seems responsive to phytohormones and environmental factors. RNA-seq data and RT-qPCR analysis indicate specificity to tissues different developmental stages, implying the potential involvement of *ZmMCJs* in tissue differentiation and seed development. This investigation offers valuable insights to enhance our understanding of the evolutionary mechanisms and functional characteristics of the MCJ gene family in maize.

### Supplementary Information

Supplementary Material 1.

Supplementary Material 2.

Supplementary Material 3.

Supplementary Material 4.

## Data Availability

All data generated during this study are included in this published article and its supplementary information files.
