# Supplementary material for: Genome-wide identification and analysis of monocot-specific chimeric jacalins (MCJ) genes in Maize (Zea mays L.)
Source: BMC Plant Biol. 2024 Jul 6;24:636. doi: 10.1186/s12870-024-05354-4 (PMC11227246; doi:10.1186/s12870-024-05354-4)
Supplement: Supplementary file 1 — Supplementary Material 1. [file 12870_2024_5354_MOESM1_ESM.docx]

**
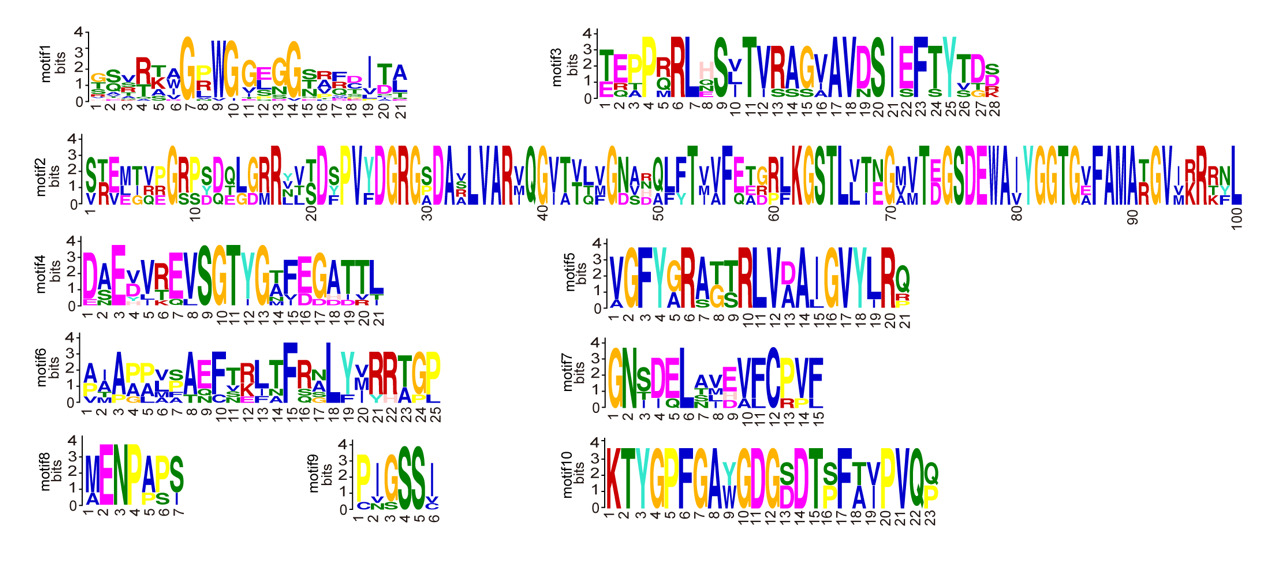
**

**Figure S1. Conserved motif logos of ZmMCJs.** They are numbered as motif 1 to motif 10, which are consistent with Figure 3B.


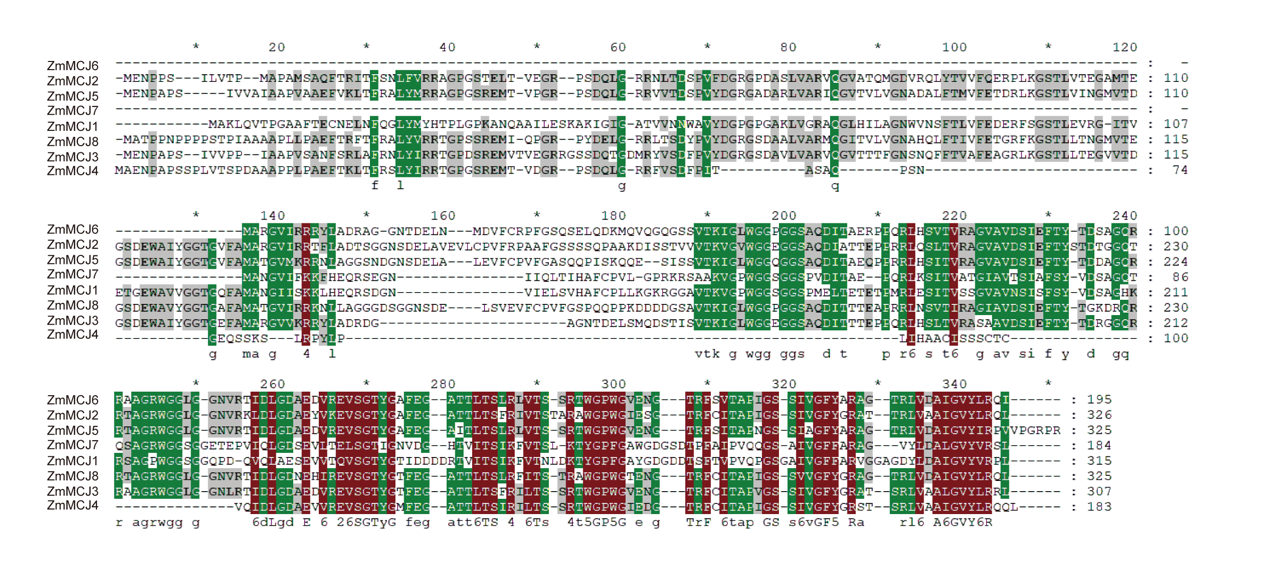


**Figure S2.** **The amino acid sequence alignment of ZmMCJs.** Amino Acid Sequence Alignment of ZmMCJs. Red shaded amino acids represent identical amino acid residues, green and grey ones indicate the similar amino acid residues.


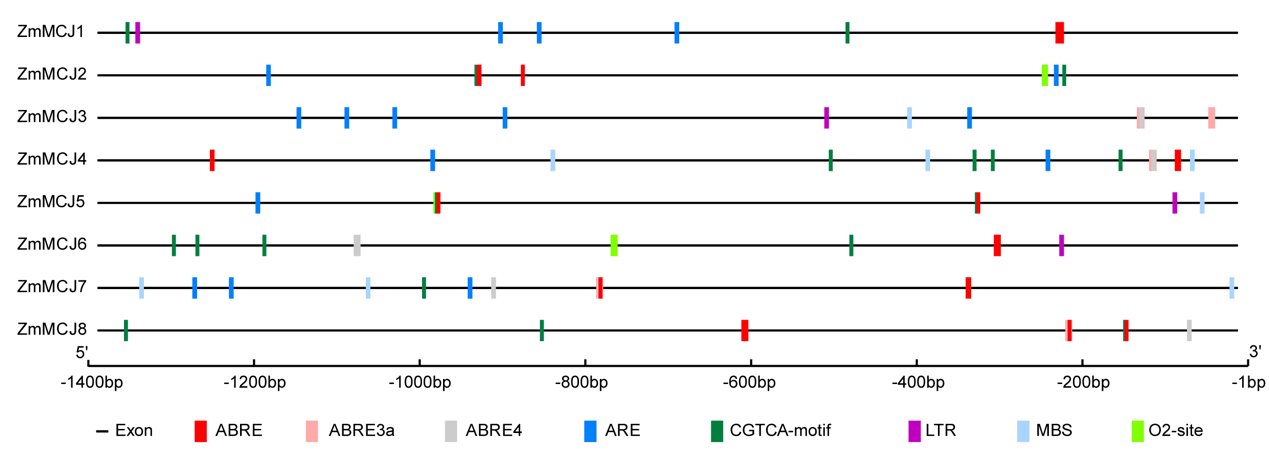


**Figure S3: Predicted *cis*-elements in ZmMCJ promoters.** Promoter region (1400 bp) of eight ZmMCJs were analyzed by PlantCARE online tool.


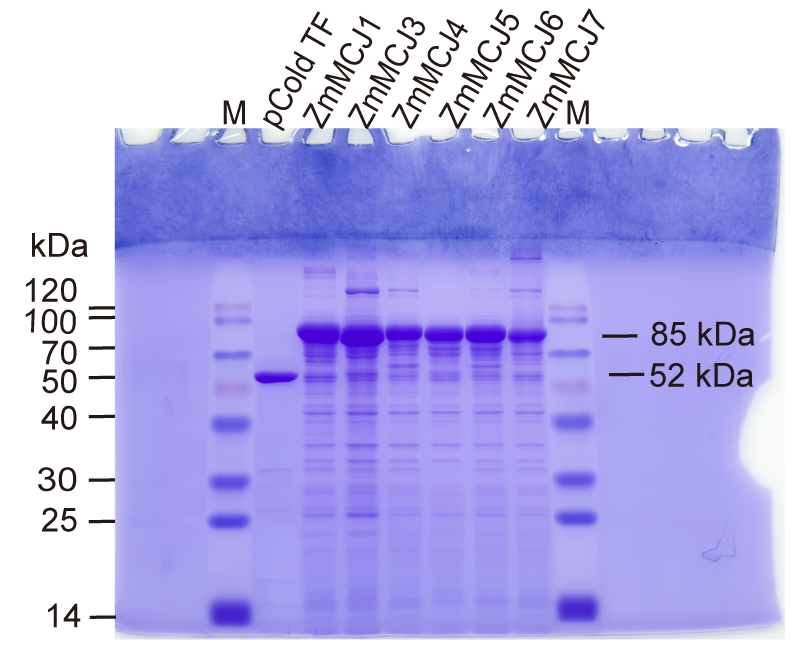


**Figure S4. Analysis of six ZmMCJ proteins expression in *Escherichia coli (E. coli)* BL21(DE3) by 12% SDS-PAGE.** Lane 1, protein molecular weight standard; lane 2, cell lysate of pCold TF/BL21(DE3) induced by isopropyl *β*-D-1-thiogalactopyranoside (IPTG) for 16 h. Lane 3 to Lane 8 are cell lysate of pColdTF-*ZmMCJs*/BL21(DE3) induced by isopropyl *β*-D-1-thiogalactopyranoside (IPTG) for 16 h.
